# Editorial: Mechanisms of lymphocyte mediated cytotoxicity in health and disease

**DOI:** 10.3389/fimmu.2023.1226870

**Published:** 2023-05-31

**Authors:** Cosima T. Baldari, Salvatore Valitutti, Michael L. Dustin

**Affiliations:** ^1^ Department of Life Sciences, University of Siena, Siena, Italy; ^2^ Institut National de la Santé et de la Recherche Médicale (INSERM) U1037, Centre de Recherche en Cancérologie de Toulouse (CRCT), Université de Toulouse III-Paul Sabatier, Toulouse, France; ^3^ Department of Pathology, Institut Universitaire du Cancer-Oncopole de Toulouse, Toulouse, France; ^4^ Kennedy Institute of Rheumatology, University of Oxford, Oxford, United Kingdom

**Keywords:** CTL, NK, cytotoxicity, immune evasion, cancer

Cytotoxic T cells (CTLs) and natural killer (NK) cells are our strongest weapon to fight viral infections and cancer, as strikingly documented by immunoregulatory disorders associated with CTL or NK cell deficiency or dysfunction. Not surprisingly, these cells are exploited for innovative immunotherapeutic strategies, including chimeric antigen receptor (CAR) T cell-based therapy. Since the initial discovery of lytic granules (LG), the killing arsenal of cytotoxic cells has expanded to include the Fas/FasL axis and, more recently, the supramolecular attack particles (SMAP), a new killing entity consisting of a lytic core surrounded by a glycoprotein shell. How these three types of weapons, and others, cooperate in space and time to kill virally infected and cancer cells is still an open question. Understanding this will provide key elements to help design highly controlled and personalized cytotoxic lymphocyte-based therapies.

Not surprisingly, viral pathogens and cancer cells have evolved counterattack strategies to disable cytotoxic cells. Elucidating these strategies, that are based on targeting the vulnerable elements of the pathway(s) of cytotoxic cell-mediated killing, is expected not only to further our understanding of the underlying mechanisms, but also to develop our own countermeasures.

In this Research Topic we present reviews, original research, methods and a case report. The collection of review articles covers recent advances on the killing mechanisms of CTLs, NK cells and also less conventional cytotoxic lymphocytes, on the strategies of resistance and counterattack of cancer cells, and on how this knowledge is being translated to the design of improved or novel immunotherapeutic strategies to enhance anti-tumor immunity. We also present two original research articles that provide new insights into the interplay of cytotoxic lymphocytes with tumor cells, two methods articles that overcome current limitations to the study of the killing machinery, and a case report of an unusual clinical presentation.

Three articles focus on CD8^+^ cytotoxic T cells. Two complementary reviews present an overview of the biogenesis and exocytosis of the lysosome-related organelles where the different weapons of the CTL killing arsenal are stored. Cassioli and Baldari review the pathways that regulate the biogenesis of the three known classes of lysosome-related organelles in CTLs and their complementary roles in the efficient and serial killing of target cells. Chang et al. describe the complex process of LG exocytosis, underscoring the similarities of this process at the immunological synapse formed by CTLs with their cellular targets and at neurological synapses, discussing how these similarities can be exploited to unravel the molecular mechanism of LG fusion with the plasma membrane. The review by Richard addresses the emerging heterogeneity of CD8^+^ T cells within what had been considered until now a single subset, discussing the phenotypical and temporal heterogeneity arising during a CD8^+^ T cell response, with a focus in the impact the strength of the TCR signal. The original research article by Lelliott et al. addresses the role of the NK cell granule protein 7 (NKG7), which is hyper-expressed in tumor-infiltrating CTLs from patients treated with immunotherapy. Using a model of CD8^+^ T cell-immunogenic colon carcinoma mouse model, they show that, unexpectedly, NKG7 knockout does not affect tumor growth despite an impairment of CTL-mediated cancer cell killing *in vitro*. However, they observe that NKG7 KO CTLs form long-lasting immune synapses with cancer cells, leading to increased secretion of TNFα, that compensates for the defect in canonical CTL toxicity by promoting TNF receptor-mediated cancer cell death.

The mechanisms of NK cell-mediated cytotoxicity, that show similarities, but also differences compared to CTLs, are discussed in two reviews. Ham et al. summarize our current understanding of the biogenesis of the NK LGs and their release, also addressing the pathways that enable NK cells to serially kill target cells. They also discuss the important issue of how NK cells protect themselves from their own cytotoxic effectors, which has been a long-standing question for all cytotoxic cells. Ramirez-Labrada et al. discuss a limitation in the studies of the mechanisms of NK-mediated cytotoxicity related to the use of purified recombinant or native proteins rather than intact NK cells, which does not allow to take into account important factors such as the influence of the stimuli received from target cells or other cellular components of the microenvironment. They review the current information of how NK cells kill target cells, discussing the different target cell death modalities that are not limited to apoptosis but also involve inflammatory pathways such as necroptosis and pyroptosis, proposing the idea that NK-mediated cell death is a new regulatory mechanism that enhances anti-cancer T cell immunity by providing inflammatory signals and tumor antigens. The original research article by Bou-Tayeh et al. provides evidence that the development and function of NK cells is altered in acute myeloid leukemia (AML). Using a mouse AML model, they show that NK cells are metabolically and functionally exhausted as the result of chronic *in vivo* IL-15/mTOR signaling as well as type I IFN signaling. The metabolic defect is recapitulated in NK cells from AML patients. Given the key role for IL-15 in NK expansion, these data provide an explanation for the NK defects previously observed in AML.

Non-conventional cytotoxic T cells are covered by two reviews. CD4^+^ T cells with cytotoxic activity have been described decades ago, however they have gained in complexity in more recent years with the expanding multiplicity of CD4^+^ T cell subsets. These cells have been identified in a number of pathological settings, including viral infections, autoimmune diseases and cancer. Cenerenti et al. present an overview of cytotoxic CD4^+^ T cells, from their discovery to the current knowledge of their killing mechanisms and their relevance to disease, with a focus on cancer and on their exploitation for immunotherapy. The review by Bolivar-Wagers et al. addresses the non-conventional role of Tregs as cytotoxic T cells, with properties shared by effector T cells. They discuss the different function of cytolytic CD4^+^ as well as CD8^+^ “effector” Tregs in the periphery, where they act as conventional T cell suppressive Tregs, and in tissues, where they exploit their killing properties for immune homeostasis. The authors also discuss the potential therapeutic exploitation of these cells for human disease, such as graft-versus-host disease.

Two reviews illustrate the mechanisms of tumor cell escape from CTL-mediated cytotoxicity. Tuomela et al. discuss the active role of the target cell in deploying resistance mechanisms throughout the execution of the death programs triggered by CTLs, highlighting these mechanisms as vulnerabilities that can be exploited by virally infected and cancer cells to evade killing. They review the mechanisms of resistance to perforin, granzyme B and death receptors. In addition to TCR-triggered killing, the authors discuss TNF-dependent cytotoxicity and the mechanisms of target cell resistance to this death program, highlighting the potential of these findings for the development of new therapeutic intervention strategies. McKenzie et al. illustrate the different early and late mechanisms of defense against CTLs deployed by tumor cells to disable the different weapons used by CTLs, namely the early release of granzymes from LGs that undergo exocytosis at the CTL immune synapse with target cells, the later multiple strategies that include enhanced autophagy, membrane repair and impairment of the death pathways, and the potential role of SMAPs in overcoming these defenses. The authors propose a multipronged strategy targeting multiple steps in the defense process to enhance the efficacy of anti-tumor immunity and immunotherapy.

The review by Espie and Donnadieu presents recent advances of the factors that underpin the effectiveness of CAR T cell-based therapies versus failure in solid tumors, discussing the importance of the strength of the adhesion of CAR T cells and cancer cells, which is determined by IFNγ and ICAM1, for cancer cell killing. They also elaborate on the translatability of these findings to improve existing CAR T cell-based therapies against solid tumors.

Two methods articles provide new tools for the study of lymphocyte-mediated cytotoxicity. Rasi et al. describe a novel mammalian expression system for the purification of high purity and biologically active recombinant LG components, that they apply to granzyme B and granulysin. This purification system could be very useful to elucidate the function of these proteins and to explore the possibility of their therapeutic application. Rudd-Schmidt et al. describe a new technology for the measurement of perforin release by murine cells during immune synapse formation. This method, involving tagging perforin at its N-terminus with a short peptide and then using tag-specific nanobodies for its detection, which overcomes the limitations precluding the exploitation of mouse disease models related to CTL defects, such as low abundance of perforin and lack of reliable antibodies.

Finally, a case report article by Zhao et al. presents a case of large granular T cell leukemia, a rare indolent leukemia associated to abnormal Fas-mediated apoptosis, with kidney involvement, characterized by circulating leukemic lymphocytes and infiltration of intra-glomerular capillaries.

Together, this Research Topic exhaustively covers the state-of-the art in lymphocyte-mediated cytotoxicity, ranging from known and new cellular players, to the pathways of biogenesis and exocytosis of the cytotoxic effectors, to the expanding mechanisms of target killing, to the defense strategies implemented by target cells. Interesting arguments are presented on how this knowledge can be translated to new therapeutic approaches aimed at enhancing the anti-cancer T cell response and improving the effectiveness of CAR T cell-based immunotherapy. We believe it will make useful reading to basic and translational scientists alike.

## Author contributions

CTB, SV and MLD drafted the manuscript. All authors contributed to the article and approved the submitted version.

